# Unveiling the genetic basis of Fusarium wilt resistance in chickpea using GWAS analysis and characterization of candidate genes

**DOI:** 10.3389/fgene.2023.1292009

**Published:** 2024-01-19

**Authors:** Alsamman M. Alsamman, Khaled H. Mousa, Tawffiq Istanbuli, Mamdouh M. Abd El-Maksoud, Sawsan Tawkaz, Aladdin Hamwieh

**Affiliations:** ^1^ International Center for Agricultural Research in the Dry Areas (ICARDA), Giza, Egypt; ^2^ Agricultural Research Center (ARC), Agricultural Genetic Engineering Research Institute (AGERI), Giza, Egypt; ^3^ International Center for Agricultural Research in the Dry Areas (ICARDA), Terbol, Lebanon; ^4^ Department of Genetics, Faculty of Agriculture, Mansoura University, Mansoura, Egypt

**Keywords:** chickpea, Fusarium wilt, SNP genotyping, genome-wide association study (GWAS), population structure

## Abstract

**Introduction:** Chickpea is a legume crop that thrives in regions with semi-arid or temperate climates. Its seeds are an excellent source of proteins, carbohydrates, and minerals, especially high-quality proteins. Chickpea cultivation faces several challenges including Fusarium wilt (FW), a major fungal disease that significantly reduces productivity.

**Methods:** In this study, a Genome-wide Association Analysis (GWAS) was conducted to identify multiple genomic loci associated with FW resistance in chickpea. We conducted a comprehensive evaluation of 180 chickpea genotypes for FW resistance across three distinct locations (Ethiopia, Tunisia, and Lebanon) during the 2-year span from 2015 to 2016. Disease infection measurements were recorded, and the wilt incidence of each genotype was calculated. We employed a set of 11,979 single nucleotide polymorphisms (SNPs) markers distributed across the entire chickpea genome for SNP genotyping. Population structure analysis was conducted to determine the genetic structure of the genotypes.

**Results and Discussion:** The population structure unveiled that the analyzed chickpea germplasm could be categorized into four sub-populations. Notably, these sub-populations displayed diverse geographic origins. The GWAS identified 11 SNPs associated with FW resistance, dispersed across the genome. Certain SNPs were consistent across trials, while others were specific to particular environments. Chromosome CA2 harbored five SNP markers, CA5 featured two, and CA4, CA6, CA7, and CA8 each had one representative marker. Four SNPs demonstrated an association with FW resistance, consistently observed across a minimum of three distinct environments. These SNPs included SNP5826041, SNP5825086, SNP11063413, SNP5825195, which located in CaFeSOD, CaS13like, CaNTAQ1, and CaAARS genes, respectively. Further investigations were conducted to gain insights into the functions of these genes and their role in FW resistance. This progress holds promise for reducing the negative impact of the disease on chickpea production.

## 1 Introduction

Chickpea (*Cicer arietinum L.*) is a highly significant grain legume that holds global prominence. It is widely cultivated and consumed, particularly in developing countries, where it serves as a crucial food source (Jukanti et al., 2012; Ullah et al., 2020). According to the United Nations Food and Agriculture Organization (FAO), chickpea is cultivated in over 50 countries, with India being the leading producer, contributing to over 70% of the global production. Pakistan and Iran, the next two largest producers, account for 10% and 5% of the total production, respectively. Other notable producing nations such as Turkey and Australia contribute 4% and 3%, respectively (Muehlbauer and Sarker, 2017). Chickpea is grown in a variety of ecological conditions, exposing it to a variety of biotic and abiotic stresses. Among the biotic stresses, Fusarium wilt (FW) stands out as a significant constraint to chickpea production (Garg et al., 2018). FW is a disease caused by the fungus *Fusarium oxysporum* f. sp. ciceri, which targets chickpea plants (Schumann and Sands, 2006). FW is a significant soil-borne disease, particularly prevalent in the Mediterranean, South Asia, and East Africa regions, where it induces root and stem rot, ultimately impacting chickpea production (Alloosh et al., 2019). In warm and dry regions, FW stands as the most destructive root disease, resulting in yield losses ranging from 10% to 40% and, in some cases, complete crop failure (Garg et al., 2018). Symptoms of FW include vascular clogging, stunted growth, yellowing and wilting of leaves, and eventual plant death (Okungbowa and Shittu, 2012; Leitão et al., 2020). The fungus primarily infects plants through their roots, but it can also spread through contaminated soil or seeds (Singh and Sharma, 1997; Fan et al., 2022).

*F. oxysporum* is a root-inhabiting (soil invader) fungus that reproduces asexually (Jiménez-Díaz et al., 2015). It has the ability to persist in soil for extended periods and produces toxins that impair plant function, including water and nutrient transport (Singh and Singh, 2012). The fungus invades the vascular system of chickpea plants, leading to wilting and eventual death (Singh and Vyas, 2021). Chemicals are widely used to manage FW in chickpeas; however, their usage is neither environmentally friendly nor cost-effective. Additionally, improper chemical use can harm beneficial soil microorganisms (Maitlo et al., 2019). Hence, the most effective management approach for FW in chickpeas involves integrated disease management strategies, and the development of chickpea resistant genotypes (Singh et al., 2015; Soltanzadeh et al., 2016). Modern molecular techniques such as SNP genotyping can be used to achieve this goal (Uffelmann et al., 2021).

Genome-wide association study (GWAS) have gained increasing importance in plant science, enabling researchers to identify and comprehend the genetic basis of various traits (Alseekh et al., 2021; Tibbs Cortes et al., 2021). One of the main advantages of GWAS is its applicability to a wide range important traits, including disease resistance. For instance, GWAS has successfully identified genetic markers associated with resistance to diseases. In maize affected by Fusarium ear rot, many differentially expressed genes enriched in pathways related to plant immune responses, including plant hormone signal transduction, phenylpropanoid biosynthesis, and cytochrome P450 metabolism, were detected (Yao et al., 2020). And late blight in potatoes: 18 genes associated with blight resistance were identified, among them, PGSC0003DMG400028682 encodes chitinase 1, which is related to immune response, PGSC0003DMG400036902 encodes a glycine-rich protein involved in the defence ability of the cell wall and PGSC0003DMG400031878 encode NBS-LRR resistance proteins, which are directly related to potato late blight resistance (Wang et al., 2020). By identifying genetic markers associated with disease resistance, breeders can develop cultivars that are resistant to specific diseases, thus reducing their impact on crop yields and productivity (Bartoli and Roux, 2017). GWAS can also identify specific genetic markers associated with other important traits such as yield, quality, and stress tolerance (Tibbs Cortes et al., 2021). The application of GWAS in chickpea breeding holds great potential for significantly improving crop productivity and resistance to diseases such as *F. oxysporum* infection (Sharma et al., 2016; Li et al., 2018; Varshney et al., 2019). By identifying and selecting for infection-tolerant genes and genotypes (Alseekh et al., 2021; Tibbs Cortes et al., 2021), breeders can develop cultivars with enhanced resistance to *F. oxysporum*, thereby mitigating the impact of this disease on chickpea production. This is particularly crucial in developing countries where chickpeas play a vital role in food security and livelihoods (Karaca et al., 2019).

SNP markers which are highly significant, and remain underutilized in molecular breeding programs (Tyagi et al., 2019; Fayaz et al., 2021). SNP markers are the preferred choice due to their wide genome coverage, co-dominant inheritance, chromosome-specific location, cost-effectiveness, and rapid tracking capability (Farahani et al., 2019). The present study was designed to study the population structure of a set of international chickpea germplasms. These germplasms were genotyped using SNP technology to identify potential genomic loci and genes associated with FW resistance in chickpea using GWAS. The identified SNP markers could serve as a basis for marker assisted selection and provide insights into the genetic mechanisms underlying chickpea resistance.

## 2 Materials and methods

### 2.1 Field experiment

One hundred and eighty chickpea genotypes were selected from the genetic resource section (GRS) of ICARDA based on the passport data using the focused identification of germplasm strategy (FIGS) protocol (Ahmed et al., 2021) (Supplementary Table S1). The experiment was conducted with an Alpha Lattice design in three locations: Terbol-Lebanon and Beja-Tunisia during the 2015 and 2016 seasons, and only in the 2016 season in Debre Zeit-Ethiopia. The susceptible chickpea genotype (ILC482) (Arfaoui et al., 2006) was planted every ten rows in the experimental setup. The genotypes under study were sown in rows measuring 250 cm in length, with 45 cm between each row. Two replications were maintained for all locations. Disease infection measurements were recorded for each genotype at 40 and 75 days after cultivation. The wilted plants of test entries were observed and recorded at various stages, specifically when the susceptible check had reached its peak susceptibility. The second stage data on wilted plants were recorded at the beginning of physiological maturity. Plots were scored (% of wilted or dead plants per plot). For data analysis, the maximum score was utilized. The wilt incidence of each test entry was calculated using the following formula:

$$\text{Wilt incidence}=\frac{\text{Number of wilted plants}}{\text{Total number of plants}}\times 100$$

The resistance and susceptibility levels of each test line were determined using a 1-9 rating scale (Iqbal et al., 1993). According to this scale, a rating of 1 indicates high resistance (0%–10% of plants wilted), 3- resistance (11%–20% plant mortality), 5- moderate resistance (21%–30% mortality), 7- susceptibility (31%–50% mortality), and 9- high susceptibility (more than 50% mortality). The analysis of variance was performed using Genstat software, a widely employed statistical package for data analysis and statistical modeling. The means generated across replicates were subsequently utilized in the GWAS analysis.

### 2.2 DNA isolation and SNP genotyping

DNA was extracted from the young leaves of 180 chickpea genotypes aged 4–6 weeks using the cetyltrimethylammonium bromide (CTAB) method. In summary, fresh seedling leaf was dried and ground into a fine powder. Following that, we added the powder in a 2 mL Eppendorf tube with 1 mL pre-warmed 2X CTAB buffer 2% CTAB, 0.1 M Tris HCl (pH 8.0), 1.4 M NaCl, 20 mM Ethylenediaminetetraacetic acid (EDTA). The suspension was mixed and incubated for 30 min at 65°C. After cooling for 5 min at room temperature (RT), 1 mL chloroform-isoamyl alcohol (24:1) was added to the tube and gently mixed for 10 min by shaking. The suspension was centrifuged for 20 min at RT at 4,500 rpm (Beckmann YA-12), and the supernatant was transferred to a new tube. The DNA was precipitated in 1 mL of cold isopropanol. The DNA was placed in a micro-centrifuge tube and washed twice for 20 min with a washing buffer (70% ethanol and 200 mM sodium acetate). After about 10–20 min of air drying, the DNA was dissolved in 200 *μ* of 1X TE buffer 10 mM Tris HCl, pH 8.0, 1 mM EDTA.

The extracted DNA samples were sent to Triticarte Pty. Ltd. Australia (http://www.triticarte.com.au) for SNP marker genotyping. A total of 11,979 SNP markers (Supplementary Tables S2, S3) were obtained. The SNP markers were filtered based on the following call rates greater than 80%, and minor allele frequency (MAF) ≥ 5%. After filtration 1,715 SNPs markers were accepted for data analysis.

### 2.3 Population structure

We conducted a population structure analysis to identify clusters and gain insights into the genetic makeup of 180 chickpea genotypes. The LEA R package (Gain and François, 2021) was used for population structure. The analytical process commenced by utilizing the R function snmf to evaluate the genetic structure of the population. This involved a range of potential genetic groupings (K) from 1 to 10, each repeated ten times for robustness with 10,000 iterations. The cross-entropy criterion was employed to identify the optimal number of population groups that best elucidated the genotypic data (Frichot and François, 2015; François, 2016). The results of this analysis were visualized using a barplot generated with the R ggplot2 package, illustrating the relationship with the country of origin for the genotypes.

### 2.4 Genome-wide association analysis and gene annotation

GWAS was carried out with the aim of identifying genetic markers linked to FW resistance. The phenotypic data encompassing the levels of resistance and susceptibility to FW, alongside the SNP genotyping data, were subjected to analysis using the vcf2gwas software (Vogt et al., 2022). The GWAS was conducted through the application of the mixed linear model (MLM), integrating both population structure (Q) and polygenic (K) factors, constituting the Q + K model (Wang et al., 2016). This modeling approach has gained widespread acceptance in GWAS due to its efficacy in mitigating the influence of numerous minor genetic effects and rectifying biases arising from population stratification (Wen et al., 2018). To enhance caution in our analysis, principal component analysis (PCA) was employed as a covariate. This step was taken to more thoroughly address potential confounding factors that could influence the outcomes of our study. Furthermore, an assessment using the Q-Q plot was performed, leveraging its capability to visually juxtapose the empirical data distribution against a theoretical probability distribution (Das and Resnick, 2008). To discern meaningful associations, we implemented two levels of thresholds. The initial criterion was set at a False Discovery Rate (FDR) of less than 0.1. Additionally, we adopted a *p*-value threshold of -log10-pvalue ≥2.3 (equivalent to *p*-value ≤0.005). Notably, we considered associations as significant only if they consistently manifested across a minimum of two distinct environments. This dual-threshold approach ensures a stringent and reliable identification of substantial associations in our study. We employed boxplot analysis to show the impact of each allele value on the expression of the studied phenotype. To uncover the genetic basis of FW resistance, we used the NCBI BLASTN tool to map marker sequences linked to FW-associated SNPs onto the chickpea genome, aiming to identify potential resistance genes (Varshney et al., 2013).

## 3 Results

### 3.1 Population genetic structure

**FIGURE 1:**
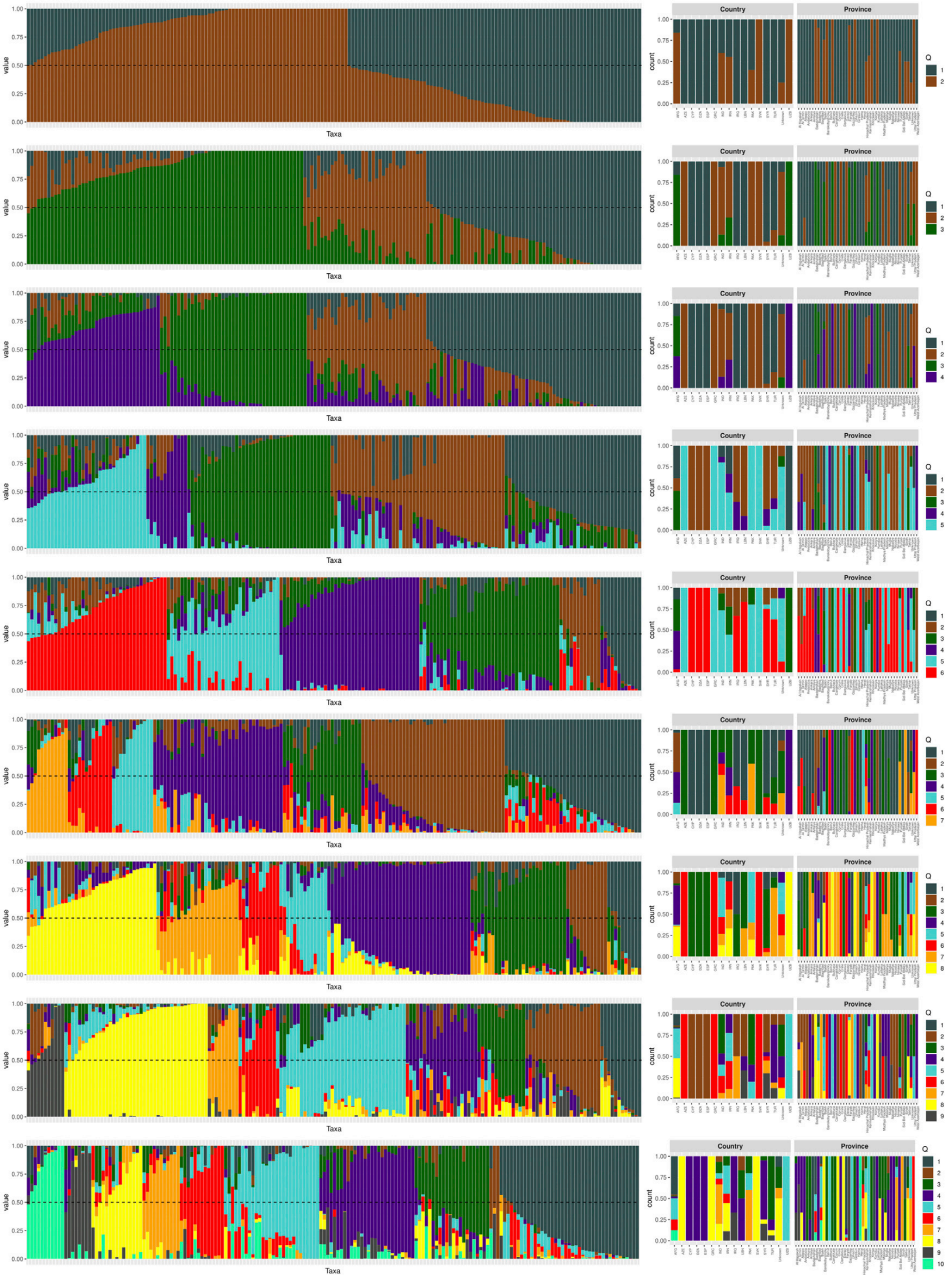
Population structure plot of the 180 genotypes. The structural analysis revealed that the optimal number of clusters, denoted as K, is 4. In the plot, each vertical bar represents an individual genotype, and the colors indicate the country of origin for each genotype.

The analysis of population genetic structure is commonly used to demonstrate the genetic similarity between populations categorized based on their geographic location (Figure 1). The structural analysis determined the optimal number of clusters as *K* = 4. This analysis revealed that the analyzed chickpea germplasm can be classified into four populations (Figure 1). The first population (Q1) consists of Cyprus, Algeria, Spain, Iraq, and Lebanon. The second group (Q2) includes Azerbaijan, Greece, Pakistan, and Slovakia, while the third (Q3) is associated with Afghanistan. The fourth population (Q4) was identified in Afghanistan, Iran, and Uzbekistan. The first group was found to be the largest population spread across different geographical areas. Additionally, there was some genetic overlap between populations I, III, and IV in Afghanistan, indicating a mixture of genetic origins from various geographic regions.

### 3.2 Genetic markers associated with FW resistance and resistance potential genes

**FIGURE 2:**
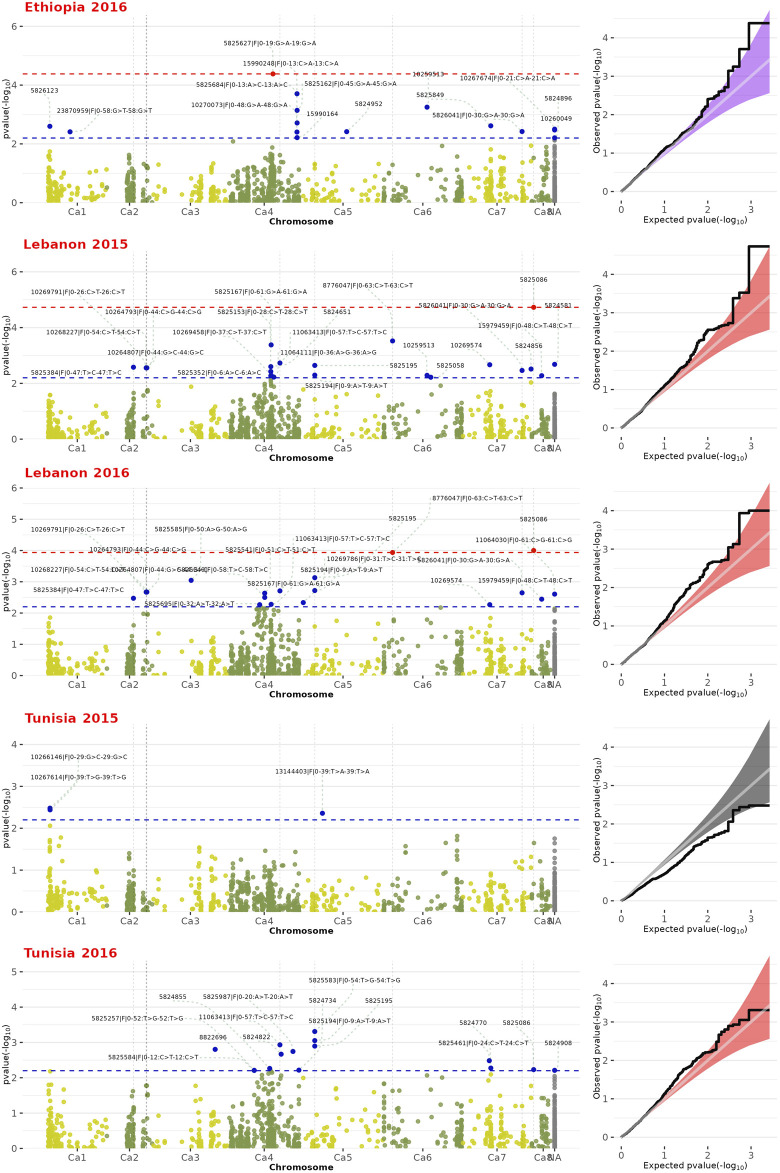
Manhattan plot and Q-Q plots depicting GWAS results for FW resistance across the chickpea genome. Red dots along the dashed red line signify significance with False Discovery Rate (FDR) ≤ 0.1. Blue dots along the dashed blue line represent SNPs associated with FW-associated resistance ≤ 0.005 (-log10-pvalue ≥ 2.3).

Initially, we employed 11,979 SNP markers for genotyping. However, only 1,715 markers met the quality control parameters for MAF and missing rate. The GWAS analysis utilized SNP genotyping data for the 180 chickpea genotypes, along with their phenotypic data replicated across three locations and 2 years. The GWAS revealed numerous SNPs associated with FW. Among these, certain SNPs were consistently observed across multiple regions, while others were specific to particular environments (Figure 2; Table 1). Three significant SNP markers associated with FW resistance, namely, SNP5825627, SNP8776047, and SNP5825086, distributed across the chickpea genome, with an FDR ≥0.1. SNP5825086, located on chromosome CA8, was detected in multiple environments Lebanon-2015 (*p*-value of 1.0 × 10^−5^) and Lebanon-2016 (*p*-value of 9.0 × 10^−5^), with an FDR ≥0.07. SNP5825627 on chromosome CA4 was exclusively identified in Ethiopia-2016 (*p*-value of 4.0 × 10^−5^) with an FDR of 0.05. Similarly, SNP8776047 on chromosome CA6 was observed in Lebanon-2016 (*p*-value of 1.0 × 10^−4^) with an FDR of 0.07 (Figure 2).

**TABLE 1:** The genetic markers associated with FW resistance across multiple studied field trials.

Rs	Ethiopia (2016)	Lebanon (2015)	Lebanon (2016)	Tunisia (2015)	Tunisia (2016)	No.
5826041	3.7 × 10^−3^	3.4 × 10^−3^	2.2 × 10^−3^			3
5825086		1.8 × 10^−5^	9.9 × 10^−5^		5.8 × 10^−3^	3
8776047		3 × 10^−4^	1.1 × 10^−4^			2
11063413		1.8 × 10^−3^	1.9 × 10^−3^		1.1 × 10^−3^	3
5825195		2.2 × 10^−3^	7.4 × 10^−4^		1.2 × 10^−3^	3
5825384		2.6 × 10^−3^	3.3 × 10^−3^			2
10268227		2.7 × 10^−3^	2.1 × 10^−3^			2
10264793		2.7 × 10^−3^	2.1 × 10^−3^			2
10269791		2.7 × 10^−3^	2.1 × 10^−3^			2
10264807		2.7 × 10^−3^	2.1 × 10^−3^			2
5825194			1.9 × 10^−3^		8.8 × 10^−4^	2

**FIGURE 3:**
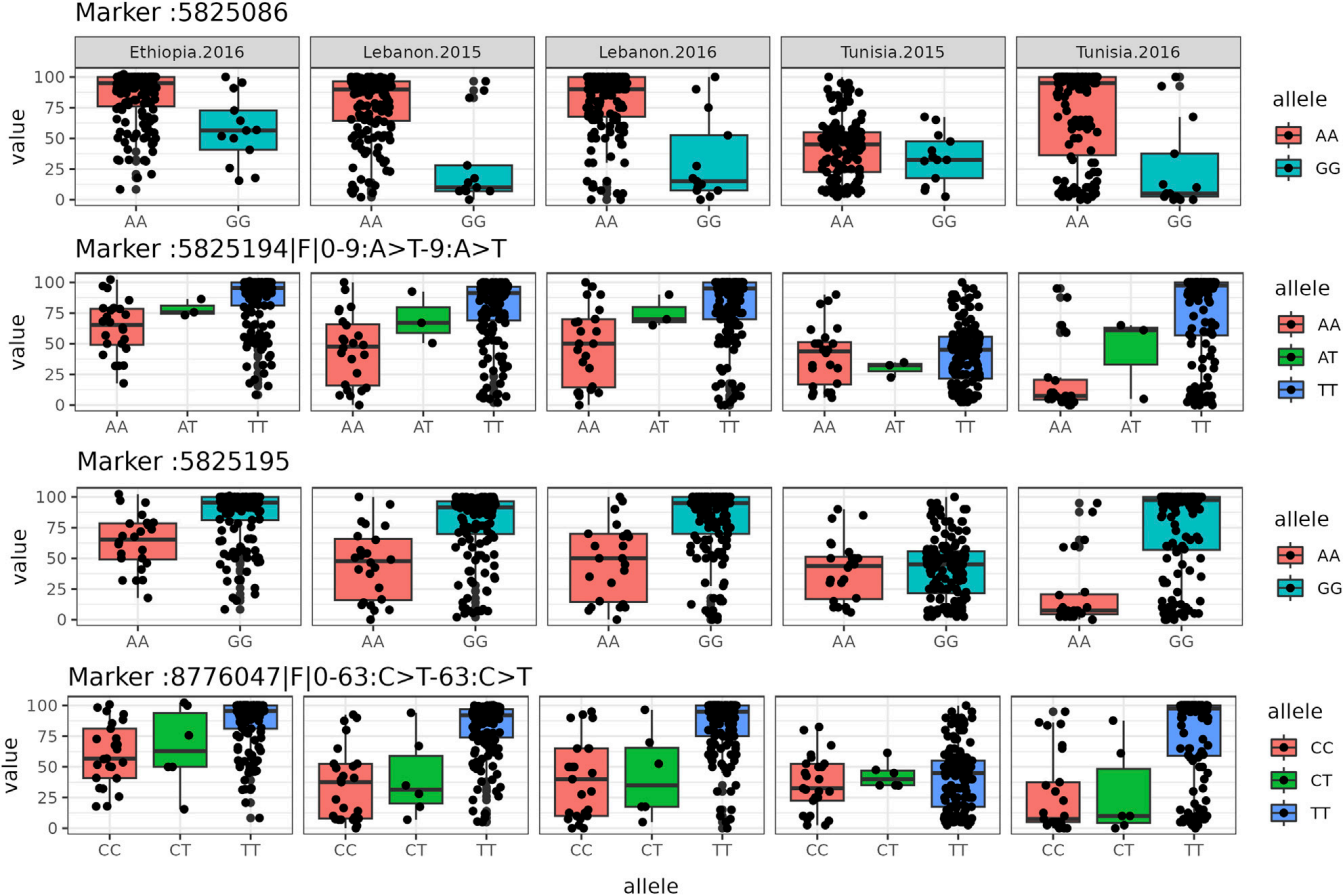
Boxplot diagrams illustrate the allelic effects of specific SNPs. In each box plot, the box represents the lower quartile, median, and upper quartile values, while black dots indicate genotypes.

**FIGURE 4:**
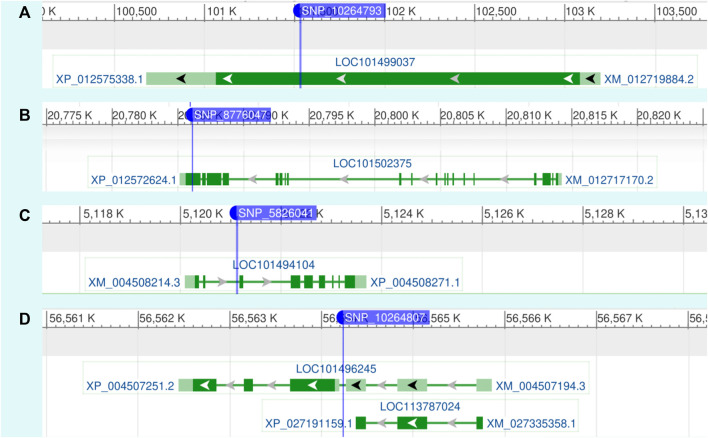
Spatial depiction of FW resistance-associated SNPs within the chickpea genome, illustrating their positions in relation to the gene structure. Introns are represented by green lines, while exons are depicted as green boxes. The depiction represents genes adjacent to SNPs **(A)** 10264793, **(B)** 8776047, **(C)** 5826041, and **(D)** 10264807, which are associated with chickpea resistance to FW in the current study.

Moreover, we identified 11 SNPs markers significantly associated with FW resistance, each with a *p*-value of ≤ 0.005. These markers were dispersed throughout the genome and across diverse environments. Notably, chromosome CA2 harbored five SNP markers (SNP5825384, SNP10268227, SNP10264793, SNP10269791, and SNP10264807), while chromosome CA5 featured two (SNP5825195 and SNP5825194). Additionally, chromosomes CA4, CA6, CA7, and CA8 each exhibited a single representative SNP marker: SNP11063413, SNP8776047, SNP5826041, and SNP5825086, respectively. Among these SNPs, some exhibited shared occurrences across multiple environments. Specifically, SNP5826041 on chromosome CA7 was consistently detected in various environments (Ethiopia-2016, Lebanon-2015, and Lebanon-2016), with a *p*-value $\geq 3.7\times 10^{-3}$ . SNPs SNP11063413 on chromosome CA4, SNP5825195 on chromosome CA5, and SNP5825086 on chromosome CA8 were identified as shared across multiple environments (Lebanon-2015, Lebanon-2016, Tunisia-2016), with a *p*-value $\geq 5.8\times 10^{-3}$ . Lastly, SNPs SNP5825384, SNP10268227, SNP10264793, SNP10269791, and SNP10264807 on chromosome CA2, along with SNP8776047 on chromosome CA6, were also identified as shared across multiple environments (Lebanon-2015 and Lebanon-2016), with a *p*-value $\geq 3.3\times 10^{-3}$ . Meanwhile, SNP5825194 on chromosome CA5 was exclusively identified in Lebanon-2016 and Tunisia-2016, exhibiting a *p*-value $\geq 1.9\times 10^{-3}$ (Figure 2; Table 1). We evaluated the influence of different SNPs on Fusarium resistance through boxplot analysis (Figure 3). Distinct differences in mean resistance scores between alleles are noticeable, especially at SNP5825086, SNP5825194, SNP5825195, and SNP8776047, revealing significant variation across various environments, as depicted in Figure 3. The results of the GWAS analysis revealed the presence of highly significant SNPs that are consistently shared across multiple regions. We meticulously mapped several SNP markers associated with FW resistance to their corresponding genes on the chickpea genome, as summarized in Table 2. Notably, these SNPs were found to be located within several genes of paramount importance, including *Endo-1,3-* *β* *-glucanase*, *RPS4B*, *SODs*, and *trpB2* as depicted in Figure 4.

**TABLE 2:** SNP marker sequence, genomic information and genes associated with fusarium resistance in chickpea.

SNPs	Marker sequence	Chr	Position	Gene names
5826041	TGC​AGT​ATT​CAA​TAG​GCA​AA	CA7	48919355	superoxide dismutase (CaFeSOD)
	CTT​CAA​AAC​TGA​GCA​TGT​TT			
	TTC​CAG​CCA​ATG​AAA​CAA​AG			
	TAAAAGGTC			
5825086	TGC​AGT​TTA​TAA​CCT​TGT​AA	CA8	2128548	40S ribosomal protein S13-like (CaS13like)
	ATC​ACA​AGT​GAT​TAC​AGA​TC			
	GGA​AGA​GCG​GTT​CAG​CAG​GA			
	ATGCCGAGA			
8776047	TGC​AGC​GTT​CCC​TCT​CGC​CC	CA6	7943702	disease resistance protein RPS4B
	AAT​TCA​GGA​CAG​TTG​AAA​AT			
	ATG​CAT​CCC​CGC​TCT​CCA​AT			
	ATTCGTCGT			
11063413	TGC​AGC​AAA​GGC​ATT​TGC​AT	CA4	42774584	protein N-terminal glutamine amidohydrolase (CaNTAQ1)
	CAA​ATA​TCA​ATC​TTG​TTA​TG			
	CTC​CTC​ATT​TAT​ACA​AAT​AA			
	AACTTTTGG			
5825195	TGC​AGA​CAT​TAC​TAA​CAC​AT	CA5	12757135	alanine–tRNA ligase (CaAARS)
	GGG​ATG​ATG​TCT​TTC​ATA​TG			
	TAT​ATA​CTT​CCA​TAA​AAG​GG			
	TAACTTGGC			
5825384	TGC​AGC​TAT​ATT​TCC​TTC​CT	CA2	21847535	FT-interacting protein 7-like
	CTA​AGT​GAT​TAT​TAT​CAA​AC			
	ACA​CAA​ATT​GTG​ATA​AAT​GT			
	ACATGGATC			
10268227	TGC​AGA​GAA​TGA​TAA​ACA​TA	CA2	32240134	uncharacterized
	TTT​CAC​GAA​TCC​AGC​GAA​AT			
	TTC​CAA​CTA​TCA​AAC​GGC​AA			
	AGAGAACAA			
10264793	TGC​AGC​ATT​GAT​GGG​AAT​GG	CA2	32255200	probable endo-1,3 (4)-beta-glucanase ARB_01444
	CAT​ATG​GTG​ATG​CTT​CAC​TT			
	GTG​ACC​ATT​GGA​TCA​ACC​TT			
	ACAGATCGG			
10269791	TGC​AGG​TGT​GAA​ACT​GTT​CT	CA2	32240200	uncharacterized
	TCA​TCC​CGG​TTT​TAC​AGA​TC			
	GGA​AGA​GCG​GTT​CAG​CAG​GA			
	ATGCCGAGA			
10264807	TGC​AGC​ATT​TTT​GGC​CTT​GG	CA2	32719559	tryptophan synthase beta chain 2
	ATG​TGA​GGT​ATG​TAT​GGG​AC			
	TGT​TGT​GAT​AAT​TAG​ATG​AA			
	GAATTTACC			
5825194	TGC​AGA​CAT​AAC​TAA​AAC​AT	CA5	12757135	alanine–tRNA ligase, chloroplastic/mitochondrial
	GGG​ATG​ATG​TCT​TTC​ATA​TG			
	TAT​ATA​CTT​CCA​TAA​AAG​GG			
	TAACTTGGC			

## 4 Discussion

Plants are permanently exposed to biotic stress, which induces changes in plant metabolism, resulting in physiological damage that ultimately reduces their productivity (Gimenez et al., 2018). Among these stresses, FW, which is a globally recognized menace, wielding substantial deleterious impacts on chickpea crop yields and quality. The genetic machinery governing FW resistance in chickpea is intricately woven, manifesting considerable genetic diversity. Notably, investigations into the genetic underpinnings, inheritance patterns, and the molecular markers underpinning FW resistance in chickpea have been conspicuously scarce (Li et al., 2021). The advent of GWAS has ushered in an era of potent tools for dissecting complex traits and genetic variations, particularly within the realm of SNP loci. These methodologies have been adeptly applied to diverse crop species in recent years, with the general linear model and mixed linear model firmly ensconced as common GWAS methodologies within the plant sciences (Wang et al., 2016; Chu et al., 2020; Thurow et al., 2020; Yuan et al., 2020; Ahmed et al., 2021; Li et al., 2021).

In this study, we assessed FW resistance across 180 chickpea genotypes in Ethiopia, Tunisia, and Lebanon during the 2015–2016 period. Utilizing SNP genotyping, we explored the genetic diversity and population structure of these accessions. Additionally, we conducted a thorough screening of the chickpea genome through GWAS to pinpoint genetic variations linked to FW resistance. The GWAS analysis revealed 11 robust SNPs with statistically significant associations to FW resistance, depicted in Figure 2. The identified 11 SNPs associated with FW resistance were distributed across the genome. Some SNPs were consistent across trials, while others were specific to certain environments. These markers were located on chromosomes CA2, CA5, CA4, CA6, CA7, and CA8. Remarkably, four SNPs consistently demonstrated associations with Fusarium wilt (FW) resistance in at least three environments—specifically, SNP5826041, SNP5825086, SNP11063413, and SNP5825195. In contrast, the remaining seven SNPs showed associations with FW in only two trials.

Guided by the reference genome sequence of chickpea, we successfully identified the genomic locations of significant SNPs in the chickpea genome. Several of these genes have been previously reported to play a role in the plant’s defense response and are associated with resistance to specific diseases or viruses. One such gene is Endo-1,3-*β*-glucanase (official name; glucan endo-1,3-*β*-d-glucosidase), which belongs to glycoside hydrolase family 17 and acts synergistically with chitinases to inhibit the growth of fungal pathogens by hydrolyzing the 1,3-*β*-glucosyl linkages (1,3-*β*-glucan and 1,3; 1,6-*β*-glucan) (Higa-Nishiyama et al., 2006; Shi et al., 2020). In plants, especially monocots, defense-associated endo-1,3-beta-glucanases (PR-2 proteins) are grouped into the highly divergent glucanase subfamily A, which also includes glucanases whose primary function is involved in plant defense, as well as in fundamental physiological processes such as germination, microspore generation, and embryogenesis (Higa-Nishiyama et al., 2006; Meirinho et al., 2010). Plants possess the remarkable ability to detect the presence of microbial organisms by utilizing components of their innate immune system. This system plays a crucial role in triggering an appropriate defense response upon pathogen invasion (Saucet et al., 2021). Recognition of pathogen effectors is a fundamental aspect of plant immunity, and it is mediated by intracellular NB-LRR immune receptors encoded by Resistance (R) genes (Saucet et al., 2015). Among these R genes, RPS4B stands out as a disease resistance R protein that specifically interacts with the *Pseudomonas* avirulence effector (AvrRps4) (Figure 4D; Table 2). The interaction between RPS4B and AvrRps4 triggers a hypersensitive response (Zhao et al., 2019). Moreover, RPS4B is capable of independently activating defense genes, thereby providing a level of resistance that is adequate in response to AvrRps4 (Saucet et al., 2015).

Abiotic and biotic stresses pose significant challenges to plant growth and productivity. When faced with stressors like pathogen attacks, plants activate a comprehensive array of genes dedicated to defensive molecular mechanisms. One crucial response is the oxidative burst, characterized by the rapid production of reactive oxygen species (ROS) (Faize et al., 2012). Plants possess robust defense mechanisms against ROS, including strategies to both limit their formation and facilitate their removal. At the forefront of this defense are superoxide dismutases (SODs), which constitute the initial line of defense against ROS (Alscher et al., 2002). These metal-containing enzymes catalyze the dismutation of superoxide radicals into oxygen and hydrogen peroxide, playing a pivotal role in defending against toxic-reduced oxygen species, generated as byproducts of numerous biological oxidations (Bowler et al., 1994). Furthermore, SODs play various roles in regulating H2O2 concentrations. This regulation is central to the expression of disease resistance in multiple pathosystems and plays a crucial role in signaling pathways that reinforce defense-related genes (Li et al., 2019). Additionally, H2O2 has been identified as a key regulator in a wide array of other physiological processes, including photosynthesis, senescence, and overall plant growth and development (Faize et al., 2012). Tryptophan synthase beta chain 2 (trpB2) is a component of the enzyme tryptophan synthase. It is an essential enzyme found in bacteria, yeasts, molds, and plants, and is involved in the biosynthesis of the amino acid tryptophan (Busch et al., 2014; Schattenkerk, 2017). Tryptophan is an essential amino acid required by animals for the production of proteins and other compounds, such as neurohormones, serotonin, and the vitamin nicotinic acid. In plants, it serves as a precursor to auxin and a variety of secondary metabolites, such as camalexin and glucosinolates, which protect plants from fungal, bacterial, and herbivore attacks (Miao et al., 2019; Liu et al., 2022).

In comparison, Channale et al. investigated the genetic basis of *P. thornei* resistance in chickpeas through GWAS. They analyzed a dataset comprising 492,849 SNPs and 278 chickpea accessions. Their study identified 24 significant QTNs associated with *P. thornei* resistance, distributed across all chickpea chromosomes. Six candidate genes, including receptor-linked kinases (RLKs) and GDSL-like Lipase/Acylhydrolase, were identified in close proximity to these QTNs. These candidate genes have been previously linked to plant-pathogen interactions (Channale et al., 2023). Similarly, Agarwal et al. focused on the genetic basis of Pythium ultimum resistance in chickpeas using GWAS. They analyzed a diverse chickpea diversity panel of 184 accessions and performed an analysis of 302,902 SNPs. Their study identified a major quantitative trait locus (QTL) and 14 candidate genes associated with resistance to Pythium ultimum. The candidate genes, including Ca_19996, Ca_09957, and Ca_22742, represent significant advancements in understanding disease resistance in chickpeas (Agarwal et al., 2022). Furthermore, Raman et al. conducted a study to evaluate the resistance to Ascochyta blight (AB) in chickpea using GWAS. They analyzed a genome-wide association set of 251 genotypes and identified twenty-six genomic regions associated with AB resistance on chromosomes Ca1, Ca4, and Ca6. Their study employed various GWAS models, including single and multi-locus mixed models and haplotyping trend regression, to identify these genomic regions (Raman et al., 2022). Overall, these studies, along with our own, highlight the effectiveness of GWAS in identifying genetic markers and candidate genes associated with various disease resistances in chickpeas. The identified genes, such as CaFeSOD, CaS13like, CaNTAQ1, CaAARS, RLKs, GDSL-like Lipase/Acylhydrolase, and others, provide valuable insights into the molecular mechanisms underlying disease resistance and can contribute to the development of breeding strategies for improving chickpea cultivars.

Our study on the genetic basis of FW resistance in chickpeas has certain limitations that can be addressed to further enhance our understanding of this trait. Firstly, the inclusion of a larger and more diverse panel of chickpea genotypes would provide a broader representation of the genetic variations associated with FW resistance. Expanding the number of genotypes in future studies could uncover additional markers and genes linked to resistance. Additionally, conducting experiments across a wider range of locations and seasons would help account for environmental variations and increase the robustness of the findings. While our study focused on SNP markers, integrating other marker types and exploring structural variations could provide a more comprehensive understanding of the genetic architecture underlying FW resistance. Furthermore, functional validation of candidate genes identified in our study would strengthen their association with resistance. Gene expression analysis and functional genomics approaches can provide insights into the molecular mechanisms and pathways involved. Despite these limitations, our study contributes valuable insights into FW resistance and lays the foundation for further research and breeding efforts. By addressing these limitations, future studies can build upon our findings, leading to improved FW resistance in chickpeas and benefiting crop production and food security.

## Data Availability

The datasets presented in this study can be found in online repositories. The names of the repository/repositories and accession number(s) can be found in the article/Supplementary Material.

## Supplementary material

SUPPLEMENTARY TABLE S1 The passport data for the studied 180 chickpea genotypes, including their country of origin and ICARDA Genbank ID.

SUPPLEMENTARY TABLE S2 The SNP genotypic data of the studied 180 chickpea genotypes.

SUPPLEMENTARY TABLE S3 The phenotypic data on FW infection from the field experiment conducted in three locations.
